# Relationship between vitamin D status, glycemic control and cardiovascular risk factors in Brazilians with type 2 diabetes mellitus

**DOI:** 10.1186/s13098-016-0188-7

**Published:** 2016-11-16

**Authors:** Maria Creusa Rolim, Bárbara Mendes Santos, Gildasio Conceição, Paulo Novis Rocha

**Affiliations:** 1 Postgraduate Program in Health Sciences, Federal University of Bahia, Salvador, Bahia Brazil; 2 Department of Medicine and Diagnostic Support, Medical School of Bahia, Federal University of Bahia, Salvador, Bahia Brazil; 3 Medical Genetics Laboratory, Federal University of Bahia, Salvador, Bahia Brazil

**Keywords:** Vitamin D, Type 2 diabetes mellitus, Obesity, Dyslipidemia

## Abstract

**Objectives:**

Determine the prevalence and identify predictors of hypovitaminosis D in patients with type 2 diabetes mellitus (T2DM); 2) correlate vitamin D levels with variables indicative of glycemic control and cardiovascular risk.

**Research design and methods:**

We conducted a cross-sectional study with consecutive patients treated at a University Hospital’s Endocrinology outpatient clinic located at 12°58′S latitude, between October 2012 and November 2013. Hypovitaminosis D was defined as 25-hydroxyvitamin D < 30 ng/mL (chemiluminescence).

**Results:**

We evaluated 108 patients with mean duration of T2DM of 14.34 ± 8.05 years and HbA1c of 9.2 ± 2.1%. Mean age was 58.29 ± 10.34 years. Most were women (72.2%), non-white (89.8%) and had hypertension (75.9%) and dyslipidemia (76.8%). Mean BMI was 28.01 ± 4.64 kg/m^2^; 75.9% were overweight. The prevalence of hypovitaminosis D was 62%. In multiple logistic regression, independent predictors of hypovitaminosis D were female gender (OR 3.10, *p* = 0.02), dyslipidemia (OR 6.50, *p* < 0.01) and obesity (OR 2.55, *p* = 0.07). In multiple linear regression, only total cholesterol (β = −0.36, *p* < 0.01) and BMI (β = −0.21, *p* = 0.04) remained associated with levels of 25-hydroxyvitamin D.

**Conclusions:**

Using currently recommended cutoffs, the prevalence of hypovitaminosis D in Brazilians with T2DM was as high as that of non-tropical regions. Female gender, dyslipidemia and obesity were predictors of hypovitaminosis D. Low levels of 25-hydroxyvitamin D were correlated with high cholesterol and BMI values. Future studies are needed to evaluate whether vitamin D replacement would improve these parameters and reduce hard cardiovascular outcomes.

## Background

Type 2 diabetes mellitus (T2DM) is a serious and growing global health problem. In 2013, there were 382 million people with diabetes; this number is estimated to grow to 592 million by 2035. The highest prevalence rates of T2DM are found in developing countries [1].

Hypovitaminosis D is an emerging health problem that affects approximately one billion people worldwide [2] and might be increasing in frequency [3]. Vitamin D is an important hormone for mineral homeostasis and bone integrity but also has several pleiotropic effects outside the skeleton, including the endocrine system [4]. Recent evidence suggests that hypovitaminosis D is common in patients with type 2 diabetes mellitus [5].

Hypovitaminosis D may be a neglected cardiovascular risk factor in patients with type 2 diabetes mellitus [6, 7] as vitamin D appears to influence several pathways that have been linked to coronary artery disease, such as inflammation, vascular calcification, proliferation of smooth muscle cells in vascular tissue, myocyte hypertrophy, arterial intimal thickness, renin-angiotensin system, blood pressure control and insulin resistance [8–10].

To further examine this issue, we sought to determine the prevalence of hypovitaminosis D in patients with type 2 diabetes mellitus and investigate the relationship between vitamin D levels and variables indicative of glycemic control and cardiovascular risk factors.

## Methods

### Study design

Observational, cross-sectional study. Patients were recruited between October 2012 and November 2013.

### Study site

Endocrinology outpatient clinic affiliated with the Hospital of the Federal University of Bahia, located in Salvador, Bahia—Brazil (latitude 12°58′S).

### Study population

Adult patients with type 2 diabetes mellitus. We did not include patients with other types of diabetes, current or previous use of vitamin D or multivitamins, pregnant women, patients with chronic kidney disease, patients with malabsorptive intestinal diseases or status post bariatric surgery and patients taking anticonvulsants, drugs for the treatment of HIV-AIDS, steroids, rifampicin, cholestyramine or orlistat.

### Clinical variables

We conducted individualized interviews to collect data regarding age, sex, self-reported race, time of diagnosis of diabetes, medications in use, and comorbidities. Height to the nearest 0.1 cm and weight to the nearest 0.1 kg were measured with participants wearing light clothing and no shoes. Participants with a body mass index (BMI) of 25.0–29.9 kg/m^2^ were classified as overweight, and those with BMI ≥ 30.0 kg/m^2^ were classified as obese.

### Laboratory tests

All patients were asked to stop oral antidiabetic drugs and insulin for at least 12 h prior to examination. Patients were also asked to refrain from strenuous physical exercise and alcohol intake during 3 days and fast for at least 10 h before the serum tests. Blood samples were processed for storage within 24 h of collection. Serum 25-hydroxyvitamin D [25(OH)D] was determined using a chemiluminescence kit (DiaSorin LIAISON, MN, USA) that recognizes both vitamin D2 and D3 equally. Two levels of controls provided by the manufacturer were run in each assay. Inter- and intra-assay coefficients of variation based on repeated analysis of a pooled control were 15 and 13%, respectively. The lower limit of detection of the assay was 2.8 ng/mL. Levels of 25-hydroxyvitamin D were stratified according to the classification of the Endocrine Society 2011 [11]: deficiency (<20 ng/mL), insufficiency (20–29 ng/mL) and sufficiency (≥30 ng/mL). Serum glycated hemoglobin (HbA1c) was determined using HPLC assay on automated analyzer. Serum calcium, phosphorus, alkaline phosphatase, parathyroid hormone, creatinine, total cholesterol, high-density lipoprotein-cholesterol (HDL-c), low-density lipoprotein-cholesterol (LDL-c), triglycerides, uric acid, ultra-sensible C-reactive protein (CRP), glucose concentrations and microalbuminuria were measured using standardized and automated assays.

### Sample size

Sample size was calculated using the software OpenEpi (http://www.openepi.com). The minimum estimated sample size to detect a prevalence of hypovitaminosis D of 50%, with a precision of 10–95% confidence limits was 97 patients.

### Statistical analyses

Continuous data were presented as mean (±standard deviation) or median (inter-quartile range). Categorical variables were summarized using absolute and relative frequencies and compared using the Chi-square test. The relationship between continuous variables indicative of glycemic control and cardiovascular risk factors with 25(OH)D levels was evaluated using Pearson’s correlation analyses. Subsequently, variables that were correlated with 25(OH)D with *p* < 0.10 were entered into a backward multiple linear regression model (with vitamin D level as the dependent variable). A similar strategy was used to identify predictors of hypovitaminosis D (a binary event) by logistic regression. Variables that were associated with hypovitaminosis D (*p* < 0.10) on univariate analyses were entered into a backward, multivariate logistic regression model. A two-sided *p* value <0.05 in final analyses was deemed significant. All statistical analyses were performed using IBM SPSS for Windows version 20.0 (SPSS Inc., Chicago, IL).

## Results

### Characteristics of the study population

We recruited 108 subjects with type 2 diabetes mellitus. Mean age was 58.29 ± 10.34 years and the majority were women (72.2%) and non-white (89.8%). The duration of type 2 diabetes diagnosis was 14.34 ± 8.05 years (Table 1); of note, 89.8% of the patients had had type 2 diabetes mellitus for 5 years or longer.

**Table 1:** Demographic and clinical characteristics of 108 T2DM patients followed at an outpatient Endocrinology Clinic in Brazil

Variable		Overall	Hypovitaminosis D		*p*
		(n = 108)	No (n = 41)	Yes (n = 67)	
Age (years)		58.29 ± 10.34	58.24 ± 9.99	59.19 ± 10.33	0.48
Female gender		78 (72.2%)	23 (56.1%)	55 (82.2%)	*0.01*
Non-white skin color		97 (89.8%)	39 (95.1%)	58 (86.6%)	0.20
T2DM duration (years)		14.34 ± 8.05	15.38 ± 8.30	14.06 ± 8.30	0.59
BMI		28.01 ± 4.64	27.17 ± 3.87	28.65 ± 4.99	0.13
Obesity ^a^		35 (32.4%)	8 (19.5%)	27 (40.1%)	*0.02*
Comorbidities	Hypertension	82 (74.1%)	30 (73.2%)	52 (77.6%)	0.60
	Dyslipidemia	83 (76.8%)	23 (56.1%)	60 (89.5%)	<*0.01*
Insulin use	Insulin use	78 (72.2%)	30 (73.2%)	48 (71.6%)	0.86
	NPH insulin	77 (71.3%)	29 (70.7%)	48 (71.6%)	0.92
	Regular insulin	42 (38.9%)	18 (43.9%)	24 (35.8%)	0.40
Oral antidiabetic agent	Sulfonylurea	26 (24.1%)	10 (24.4%)	16 (23.9%)	0.95
	Metformin	84 (77.8%)	34 (82.9%)	50 (74.6%)	0.31
	α-Glucosidase inhibitor	5 (4.6%)	1 (2.4%)	4 (6.0%)	0.40
Combination treatment	Insulin plus OAD	57 (52.8%)	24 (58.5%)	33 (49.2%)	0.35
	Insulin plus metformin	5 (4.6%)	2 (4.9%)	3 (4.5%)	1.00
	Insulin plus sulfonylurea	56 (51.8%)	24 (58.5%)	32 (47.8%)	0.28
	Metformin plus sulfonylurea	23 (21.3%)	9 (21.9%)	14 (20.9%)	0.90
Lipid lowering agents	Statins	69 (63.9%)	20 (48.8%)	49 (73.1%)	*0.01*
	Fibrates	4 (3.8%)	1 (2.4%)	3 (4.5%)	1.00
Antihypertensive agents		86 (79.6%)	33 (80.5%)	53 (79.1%)	0.86

Italic values indicate statistically significant *p*-value

*T2DM* type 2 diabetes mellitus, *OAD* oral antidiabetic agent

^a^BMI ≥30 kg/m^2^

Most patients were overweight or obese (75%). Mean BMI was 28.1 ± 4.6 kg/m^2^ (Table 1). Women had significantly higher BMI values than men (28.57 ± 4.75 kg/m^2^ vs. 26.14 ± 3.67 kg/m^2^; *p* = 0.01).

Most patients used insulin (72.2%), with a mean dose of 0.52 ± 0.28 IU/kg. Only NPH and regular insulin were used. Mean doses were: NPH 29.45 ± 14.20 IU (8.0 to 84.0) and regular insulin 13.74 ± 7.37 IU (4.0–36.0). The most commonly used oral antidiabetic agent was metformin (77.8%). The most common combinations were metformin plus insulin and metformin plus sulphonylurea (Table 1).

The main comorbidities associated with type 2 diabetes mellitus were hypertension (74.1%) and dyslipidemia (76.8%) (Table 1).

### Prevalence of and risk factors for hypovitaminosis D

**Fig. 1:**
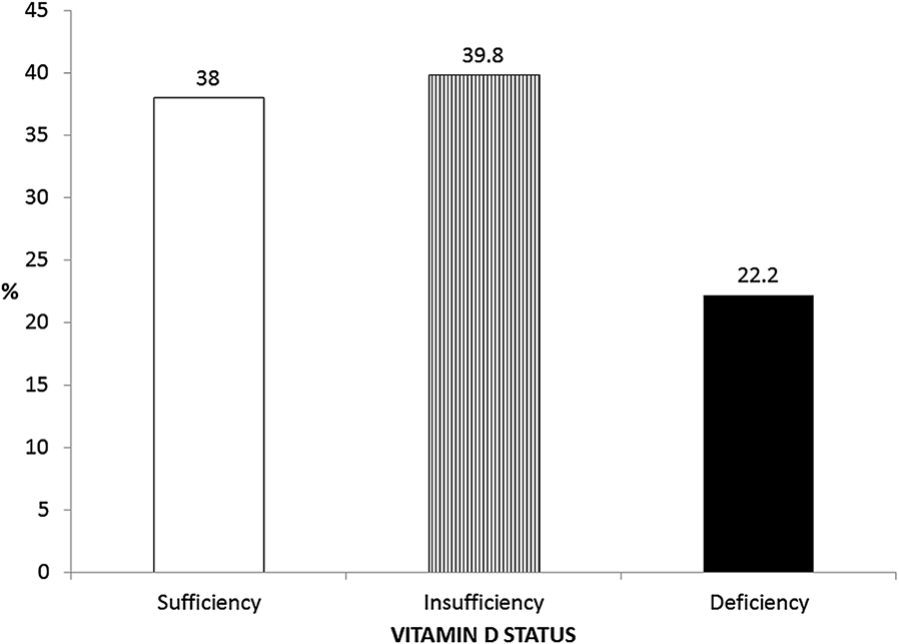
Vitamin D status of 108 T2DM patients followed at an outpatient Endocrinology Clinic in Brazil

**Fig. 2:**
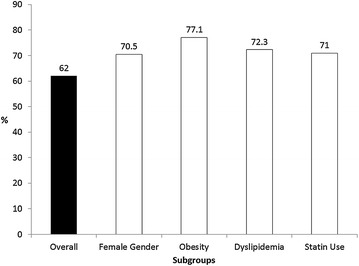
Prevalence of hypovitaminosis D: overall and stratified into subgroups with significantly higher prevalence

Mean 25(OH)D level was 28.10 ± 9.26 ng/mL. The overall prevalence of hypovitaminosis D was 62% (39.8% insufficient and 22.2% deficient) (Fig. 1). Hypovitaminosis D was more common among women (70.5 vs. 40%, female vs. male, *p* = 0.01), obese (77.1 vs. 54.2%, obese vs. non-obese, *p* = 0.02), dyslipidemic (72.3 vs. 28%, dyslipidemic vs. non-dyslipidemic, *p* < 0.01) and statin users (71% vs. 46.1%, users vs. non-users, *p* = 0.01) (Table 1; Fig. 2).

We conducted univariate logistic regression analyses to identify potential predictors of hypovitaminosis D [defined by a cutoff of 25(OH)D <30 ng/mL]. Gender, history of dyslipidemia, statin use and obesity were entered into the model but only female gender (OR 3.10, *p* = 0.02), dyslipidemia (OR 6.50, *p* < 0.01) and obesity (OR 2.55, *p* = 0.07) emerged as independent predictors of hypovitaminosis D (Table 2).

**Table 2:** Univariate and multivariate backward logistic regression analyses to identify independent predictors of hypovitaminosis D

Variable	Univariate	*p*	Multivariate	*p*
	OR (95% CI)		Adjusted OR (95% CI)	
Female gender	3.59 (1.49–8.63)	0.00	3.10 (1.16–8.29)	0.02
Dyslipidemia	6.71 (2.48–18.17)	0.00	6.50 (2.24–18.86)	<0.01
Statin use	2.86 (1.26–6.47)	0.01		
Obesity	2.86 (1.14–7.13)	0.02	2.55 (0.92–7.06)	0.07

All 4 variables were entered into the multivariate backward logistic regression model but statin use was removed by the system on the final step

*Dyslipidemia* history of dyslipidemia, *Obesity* BMI ≥ 30 kg/m^2^

**Fig. 3:**
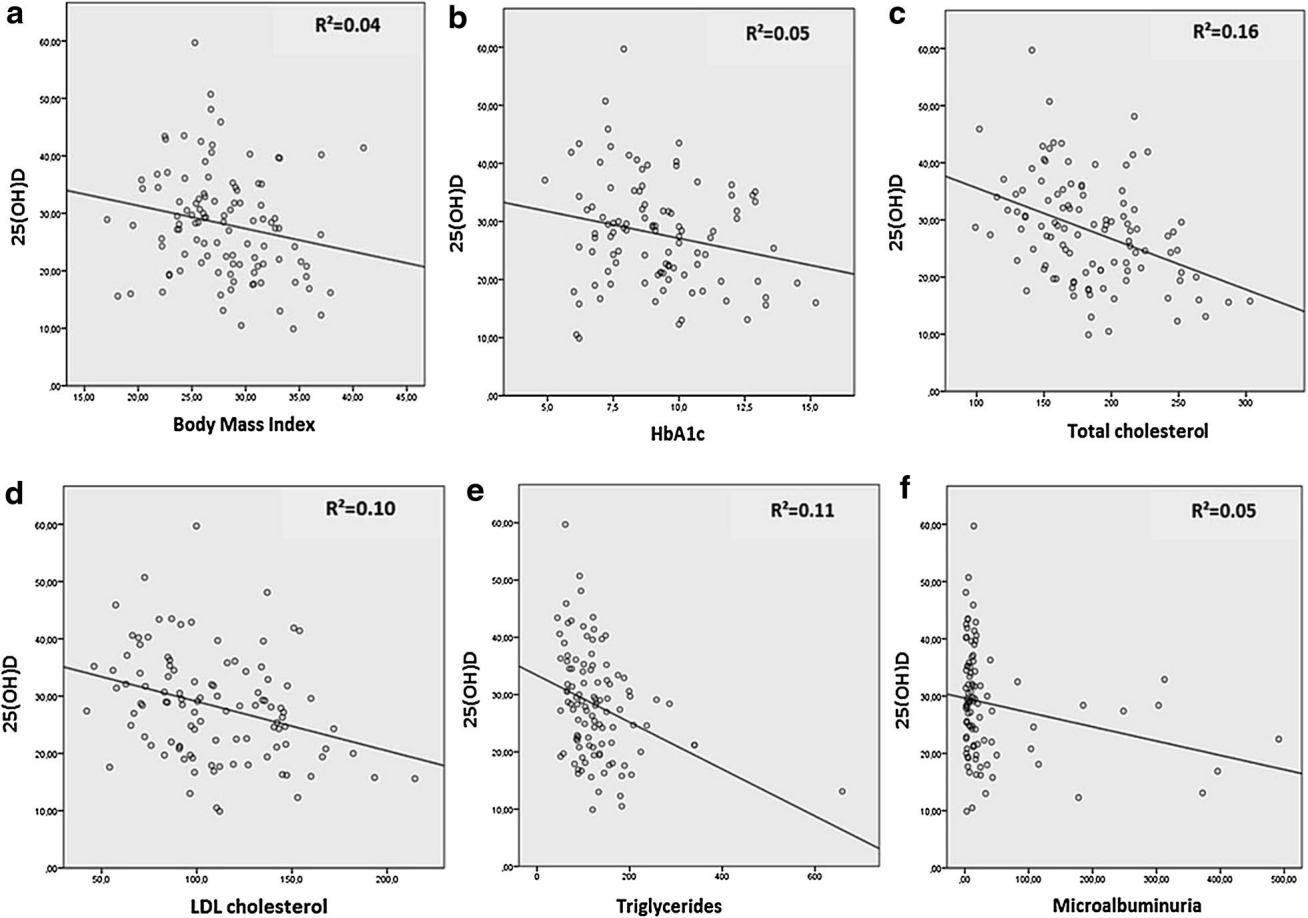
Simple linear correlation between 25-hydroxyvitamin D and variables indicative of glycemic control and cardiovascular risk. *25(OH)D* 25-hydroxyvitamin D, *HbA1c* glycated hemoglobin, *LDL* cholesterol low-density lipoprotein-cholesterol

Since the 25(OH)D cutoff used to define vitamin D deficiency in our logistic regression analyses might be the subject of some controversy, we conducted correlation and multiple linear regression analyses to further investigate the association between vitamin D levels, glycemic control and cardiovascular risk factors. There were no associations between vitamin D levels with duration of diabetes, blood pressure, fasting glucose, HDL-c, ultra-sensible CRP, uric acid, estimated glomerular filtration rate, nor with blood concentrations of calcium, phosphorus, alkaline phosphatase and PTH. The following variables showed significant inverse linear correlations with vitamin D levels: BMI (r = −0.20, *p* = 0.04), HbA1c (r = −0.22, *p* = 0.03), total cholesterol (r = −0.39, *p* < 0.01), LDL-c (r = −0.32, *p* < 0.01), triglycerides (r = −0.34, *p* < 0.01) and microalbuminuria (r = −0.23, *p* = 0.02) (Table 3; Fig. 3). Since total cholesterol and LDL-c were highly correlated with each other (r = 0.932, p < 0.01), we chose to include only one of these two explanatory variables in the final multiple linear regression model to avoid colinearity. We selected total cholesterol because its linear correlation with vitamin D levels was stronger than that of LDL-c. These variables were then entered into a backward, multiple linear regression model (with vitamin D level as the independent variable); only total cholesterol (unstandardized β coefficient = −0.09, *p* < 0.01) and BMI (unstandardized β coefficient = −0.41, *p* = 0.04) remained independently associated with levels of 25(OH)D (Table 3).

**Table 3:** Simple linear correlation and multiple linear regression with 25-hydroxyvitamin D levels as the dependent variable

Variables	Simple linear correlation	*p*	Multiple linear regression	*p*
	Pearson *r*		Unstandardized β coefficient	
BMI	−0.20	0.04	−0.41	0.04
HbA1c	−0.22	0.03		
Total cholesterol	−0.39	0.00	−0.09	<0.01
Triglycerides	−0.34	0.00		
Microalbuminuria	−0.23	0.02

## Discussion

There were no prior studies on the prevalence of hypovitaminosis D in patients with type 2 diabetes mellitus in Brazil. Herein, we showed that hypovitaminosis D was present in 62% of patients with type 2 diabetes and it was associated with female gender, obesity and dyslipidemia. Since the current study was conducted in Salvador—Bahia, located at 12°58′S, an area that receives adequate sunlight throughout the year, the high prevalence of hypovitaminosis D among individuals with type 2 diabetes encountered herein could be considered unexpected. In fact, our findings are comparable to those found in diabetic populations of non-tropical regions [12–14]. Certain characteristics of our sample may have contributed to the high prevalence of hypovitaminosis D.

First, our sample was comprised mostly of non-white participants, reflecting the demographics of Bahia, which is the Brazilian state with the highest percentage of non-white population (77.8%). There is evidence that melanin can hinder the penetration of sunlight on the skin and vitamin D conversion [15].

Second, most participants were overweight or obese. The prevalence of obesity was high in our population, corroborating the already established link between excess weight and type 2 diabetes [1, 16]. Several studies in diabetic patients report an association between obesity and hypovitaminosis D [14, 17, 18]. Since vitamin D can be sequestered in body fat, obese patients tend to have lower serum levels. A potential confounder is that obesity is also linked to an unhealthier lifestyle, characterized by less physical activity, less sun exposure and, hence, lower vitamin D levels and worse clinical outcomes [19].

Third, the majority of our participants were women. Hoteit et al. and van der Meer et al. [20, 21] suggested that female gender is an independent predictor of vitamin D deficiency. This finding is supported by other studies involving type 2 diabetes [14, 17, 22]. Indeed, female gender was an independent predictor of hypovitaminosis D in our logistic regression model.

Obesity varies between the sexes and between different races and is a recognized risk factor for vitamin D deficiency and type 2 diabetes [11, 23–27]. In our study, the women had BMIs greater than the men. Perhaps the excess body fat in females might have contributed to the observed relationship between gender and hypovitaminosis D.

Dyslipidemia was an independent predictor of hypovitaminosis D and total cholesterol was inversely correlated with levels of 25(OH)D even after controlling for confounders. Our data are consistent with other studies in the literature that evaluated cardiovascular risk in type 2 diabetes patients from sunny regions [17, 28]. Perhaps lipid levels might be the intervenient variable that explains the link between hypovitaminosis D and cardiovascular disease in patients with type 2 diabetes. However, the literature on this subject is not homogeneous and interventional studies have failed to demonstrate that raising the levels of vitamin D resulted in improvement in the lipid profile of type 2 diabetes patients [29–32].

The association between HbA1c and 25(OH)D is controversial. We found a significant weak negative correlation between HbA1C and 25(OH)D, similar to other previously published studies [12, 18, 23, 28, 33, 34]. However glycemic control was not associated with vitamin D when we controlled for confounding factors, as verified by Luo et al. and Al-Shoumer et al. [35, 36].

The Institute of Medicine suggests that the prevalence of hypovitaminosis D in the population has been overestimated due to improper use of a high cutoff level of 25(OH)D. This Institute alerts that concentrations of 25(OH)D above 30 ng/mL were not consistent with increased clinical benefit and calls for a consensus to determine the appropriate levels of vitamin D to avoid under and overtreatment [37]. Considering that our mean 25(OH)D was very close to 30 ng/mL, more studies are needed to evaluate the adequacy of the current vitamin D cutoffs in Brazilians with type 2 diabetes. In this study, a lower 25(OH)D cutoff for normality would certainly have resulted in a much lower prevalence of hypovitaminosis D.

Our work has important limitations. At our center, we use the chemiluminescence method Diasorin LIAISON to assess 25(OH)D status due to convenience, turnaround time and cost. Although the gold standard for dosing of vitamin D is the LC-MS/MS, several studies have shown that Diasorin LIAISON shows acceptable correlation with LC-MS/MS [38, 39]. We did not formally collect data on financial income, sun exposure time in outdoor activities or other characteristics predictive of outdoor behavior. We also don’t have data about the season that the blood sample was drawn but in Salvador-Bahia, temperatures are relatively constant, with about 2500 h of sunshine a year [40]; therefore, we believe that season did not play an important role in our findings. The observational, cross-sectional design precludes any conclusions regarding causality or even the direction of the association between vitamin D deficiency and cardiovascular risk factors. Moreover, our sample is small and of peculiar characteristics, which may limit the generalizability of our findings. The relationship between HbA1C and serum lipids with 25(OH)D levels may have been affected by the use of antidiabetic and lipid-lowering medications, as these drugs lower HbA1C and lipid levels regardless of the vitamin D status; similar studies in patients with early type 2 diabetes before initiating treatment could help clarify this relationship.

## Conclusion

In conclusion, we have identified a high prevalence (62%) of hypovitaminosis D among Brazilians with type 2 diabetes and an association between hypovitaminosis D with female gender, obesity and dyslipidemia. Our data indicates that availability of adequate sunlight alone is not sufficient for the prevention of vitamin D deficiency and raises awareness of hypovitaminosis D in the diabetic population, regardless of geographic location.

## Abbreviations

25(OH)D: 25-hydroxyvitamin D
BMI: body mass index
CRP: ultra-sensible C-reactive protein
HbA1c: glycated hemoglobin
HDL-c: high-density lipoprotein-cholesterol
LDL-c: low-density lipoprotein-cholesterol
OAD: oral antidiabetic agent
T2DM: type 2 diabetes mellitus
